# Hybrid Encryption Method for Health Monitoring Systems Based on Machine Learning

**DOI:** 10.1155/2022/7348488

**Published:** 2022-07-07

**Authors:** N. Malmurugan, S. Christalin Nelson, Majid Altuwairiqi, Hashem Alyami, Durgaprasad Gangodkar, Musaddak Maher Abdul Zahra, Simon Atuah Asakipaam

**Affiliations:** ^1^SRM Institute of Science & Technology (Tiruchirappalli Campus), SRM Nagar, Chennai Highway, Tiruchirappalli 621105, India; ^2^Department of Systemics, School of Computer Science, University of Petroleum and Studies (UPES), Bidholi, Dehradun, Uttarakhand 248007, India; ^3^Department of Computer Science, College of Computers and Information Technology, Taif University, Taif, Saudi Arabia; ^4^Computer Science & Engineering, Graphic Era Deemed to be University, Dehradun, Uttarakhand, India; ^5^Computer Techniques Engineering Department, Al-Mustaqbal University College, Hillah 51001, Iraq; ^6^Electrical and Electronics Engineering Department, Tamale Technical University, Tamale, Ghana

## Abstract

Numerous forms of disasters and vandalism can occur in transmission lines, which makes them vulnerable. As a result, the transmission pipes must be protected by a reliable monitoring system. When a wireless sensor network is built from disparate devices that are positioned at varying distances from one another, it can be used to monitor physical and environmental conditions in the surrounding environment. In addition to the built-in sensor on the exterior of a pipeline and sensors positioned to support bridge structures, wireless sensor networks have a range of other applications. Other uses include robotics, healthcare, environmental monitoring, and a variety of other areas of technology. It is feasible to use wireless sensor networks to monitor temperature and pressure, as well as leak detection and transmission line sabotage, among other applications. There are several different sorts of attacks that can be launched against wireless sensor networks. When it comes to information security in wireless sensor networks, cryptographic approaches play a critical role in ensuring the integrity of the data. Different types of cryptographic algorithms are now available for use in order to maintain network security. Specific difficulties must be addressed, though, and these are as follows: To strengthen the power of these algorithms, a unique hybrid encryption approach for monitoring energy transmission lines and increasing the security of wireless sensor networks is created in this study. While wireless sensor networks are being used to monitor transmission pipelines, the proposed hybrid encryption method ensures that data is transferred securely and promptly. The proposed method must follow three cryptographic principles: integrity, secrecy, and authenticity. All of the subtleties and underlying principles of the algorithm are explained in detail so that the algorithm can be put into action immediately after it is introduced.

## 1. Introduction

Make no mistake, Healthcare Monitoring has grown in popularity in recent years, owing to widespread Internet access. This has resulted in Healthcare Monitoring being a frequent new medium of consumers [1]. Modern technology has grown dramatically with the usage of cameras and mobile phone cameras, making them cheaper, more lightweight, and more practical than ever before. Because of advancements in the Internet and interactive media technology, a wide range of fields, including clinical consideration, satellite information, video and still picture stores, advanced legal sciences, and reconnaissance advances, are now being utilised in as much sight and sound as sound, video, and photos as well as video and still pictures [2]. As a result, programmes that can preserve and recover multimedia data are in high demand all of the time. There have been several visual and audible data storage and recovery frameworks developed to date in order to meet these requirements; among them are the following: Image recovery is the term used to describe the process of extracting semantically important photographs from a collection of images [3]. Image retrieval is the process of extracting semantically significant pictures from a large collection of photographs, which is also known as image search [4]. In object database analysis, the bulk of the work is devoted to the automated extraction of semantically meaningful information from the object text. When searching for images, consumers have just a vague idea of what they are looking for and how to find it. Even while current image recovery frameworks have made significant progress in addressing this problem, they are still unable to grasp the semantic meaning of images that are susceptible to human perception [5]. The semantic gap issue is the term given to this particular situation.

We did extensive research to get a better understanding of the expectations of and behaviours of a diverse variety of users since the major goal of the application is to be easy to use while still providing an engaging interface [6]. As a consequence of the application's design, the end user will find the application's functioning to be easy and straightforward.

It is possible to classify users into two groups based on their comprehension of the goods that best satisfy their needs. These individuals fall into two categories: those who are already acquainted with the product that will fulfil their needs and those who are still in the process of determining which product will suit their needs [7]. With the push of a button, users who are already acquainted with the product should be able to find it quickly and easily with a single click. Users who fall into this group may seek for a product by searching for it using the product name as the search query in their browser [8]. Searching for products using a keyword and then filtering the results based on various factors such as product type, manufacturer, price range, platform supported, and so on should be feasible for users who are seeking to identify which product will fulfil their needs [9].

While using the product, buyers should be able to see the whole product specification as well as many images taken at different zoom levels. In order for the user to see product reviews and ratings that have been provided by previous customers, the following information should be provided: They should be able to write their own reviews if they so want. Among other things, the specifications of a product should be printed, and the product page should be able to be shared with friends [10].

If at all feasible, the option to add a product to the shopping cart by dragging and dropping it into the shopping cart should be provided to improve the user's experience. The ability for a user to make adjustments to the goods in their shopping cart should be provided. Those who use the cart should be able to make any necessary adjustments to the quantities of products that have been added to it, as well as remove things from the cart [11]. A product should be able to be removed from a user's shopping cart by pulling the product out of the cart and dropping it outside of the shopping cart's confines.

Pop-up messages may be used to make the software more interactive by showing them when a product is added to or deleted from the shopping cart. Upon reaching a drop location and detecting the item that is likely to be dropped, the user might be informed [12]. Furthermore, people are impatient, which makes it vital for websites to load fast for them to be successful.

Additionally, I undertook substantial research into other methodologies for constructing this application and was able to include a few more powerful features into the final product as a result of this [13].

Although it is not needed, it is recommended that you use the ASP.NET controls and the AJAX Toolkit controls in your application since they improve navigation, usability, and interaction with the application.

Generally speaking, the system's viability may be divided into the following categories, which are listed alphabetically:

Considering that the sole investment necessary is the acquisition of a computer that fits the aforementioned fundamental parameters, this project is both feasible and affordable. Only the costs of gaining Internet access will be incurred by users in order to benefit from the software [14].

In addition, it provides final assurances that software meets all functional, behavioural, and performance standards that have been established. The employment of techniques such as black box testing is common.

Generally speaking, there are three primary components to consider.

As part of the validation test criteria, an examination of the software configuration is carried out in order to ensure that it is fully functional (no. in place of no. and char in place of char).

The following are the differences between alpha and beta testing: Alpha testing is performed at the developer's location (i.e., at home), while Beta testing is performed after the programme has been deployed. I was unable to join in the beta testing since my application had not yet been released to the public [15].

*Exemplifications of Test Cases*. When it came to putting the device through its paces, I used a range of test scenarios. In order to assess whether or not the appropriate output was generated, a variety of inputs were used in a variety of situations and for a variety of inputs.

Adding a new product to the shopping cart should not need the creation of a new row inside the cart. It is necessary to adjust the amount of a product in your basket when you add it to your cart from another location. The summary must be updated as soon as any alterations to the goods are made to the cart's contents [16].

Because the same page is entering data into more than one table in the database at the same time, it is required to confirm the atomicity of the transaction before continuing. A product should be able to be dragged into a cart and then added to a cart by clicking on a button on the system, and vice versa [17].

It is possible to run tests to ensure that internal operations are carried out in accordance with specification and that all internal components have been properly tested when performing white box testing because the tester is aware of the product's internal workings when performing white box testing [18]. In white box testing, logic pathways across software are validated by providing test cases that exercise specific sets of conditions and loops, which are then run by the programed logic path verification system.

White box testing allows software engineers to design test cases that guarantee that all independent paths inside a module have been attempted at least once:

- Put all logical alternatives through their paces on both the true and false sides of the spectrum
- Inspect all loops to ensure that they are exercised both at their maximum capacity and within their operating boundaries
- Verify that the internal data structure is correct and up to date by performing a validation test

Symmetric key algorithms are quick, but they have a number of drawbacks, including a key distribution difficulty, a lack of scalability, and the fact that they only give secrecy.

Asymmetric key algorithms, on the other hand, do not have such issues, although they are much slower. Because of this, hybrid cryptography is being used. Hybrid cryptography is the combination of two technologies that act in a complimentary way, with each performing a separate role. Symmetric key algorithms generate keys that are used for bulk data encryption, while asymmetric key algorithms generate keys that are utilised for automated key distribution [19].

Specific goals are defined in terms of their point-by-point scope:

- If speed is required in a secure network, the symmetric cypher is an excellent alternative.
- As a result, asymmetric cyphers are used when communicating in an unprotected network group, and hybrid systems are the best choice when security is more important than speed. Hybrid systems improve both the security and the operational speed of cryptographic algorithms, making them more secure and faster to operate [8].
- The fundamental cryptographic algorithms are highly mathematical in nature and involve many related algorithms, such as the Random number generation algorithm, the Extended Euclidean Algorithm, the Prime Number Generation algorithm, the Miller–Rabin algorithm, and others, in order to provide the actual means of cryptography [20].
- e-commerce and online activities are growing at an exponential rate, while at the same time, cybercrime and hacker operations are on the rise. As a result, when dealing with untrusted networks, security becomes more important than transaction speed.
- As a result, digital signatures have not only proven to be a crucial technolegal need for safe transactions across unsafe channels, but they have also made e-commerce more meaningful and secure. The usage of digital signatures is popular in software distribution, financial transactions, and other situations in which it is vital to identify forgery or tampering with the signature.
- There is a significant need for a graphical user interface- (GUI-) based programme that can perform encryption, decryption, digital signature creation, and verification using hash functions from the MD5 and SHA families.

## 2. Existing Study

In order to ensure that the logic of the programme was tested at every step of its development, I fed erroneous inputs into it and observed the resulting error messages at each stage of its development. Each and every one of the loops and conditional statements is submitted to boundary condition testing and validation in order to guarantee that they work appropriately [19].

Jakarta to analyse the system's performance, it was required to create virtual consumers (clients) and evaluate the system's performance using JMeter, which is an application testing tool. Also possible is the use of the tool to evaluate the performance of static and dynamic resources (files, Servlets, Perl scripts, Java Objects, Data Bases, and Queries), in addition to additional services, such as FTP servers. It is possible to simulate a significant amount of demand on a server, network, or object in order to analyse their overall performance. It may also be used to assess their overall performance under a variety of different load conditions. When a significant number of concurrent users are present, it may be used to do a graphical examination of performance as well as to analyse the behaviour of the server, script, and object [20].

It has been my responsibility to conduct performance testing in order to offer an estimate of the peak and sustained load that the application can bear, as well as the time it will take to do so. Several pages, such as the Shop Products page (which has extensive database access, business logic, and extra photos), and the Cart Details page, have been created to do this (simple page). A few samples of test results that have been captured on a screen shot are provided in the next section. Both the application (server) and JMeter were running simultaneously on the same machine throughout the testing. This test does not take into account issues such as network speed since the server and JMeter are both running on the same computer system.

Primary Source Method refers to the process of gathering, tabulating, and analyzing data. A primary source is a piece of evidence that was obtained directly or personally concerning an event, item, person, or work of art. Histories and legal documents, eyewitness accounts, findings of tests and statistics, pieces of creative writing, audio and video recordings, speeches, and art objects are all examples of primary source material.

Primary sources include interviews, surveys, fieldwork, and Internet interactions, such as e-mail, blogs, listservs, and newsgroups. Secondary sources include books, articles, and other secondary sources. In the scientific and social sciences, primary sources are often empirical studies—research in which an experiment was conducted or a direct observation was made—in which an experiment or direct observation was made. According to [19], the findings of empirical investigations are often published in academic journals or papers presented at conferences. As part of this process, the researcher obtains data from a variety of reliable sources in order to investigate a topic and then evaluates the hypothesis using statistical methods, formulas, and other tools. Following the acquisition of data, the researcher analyses and summarises the information before concluding his study project. It is a purely theoretical way of doing research.

Secondary sources describe, discuss, interpret, comment on, analyse, evaluate, summarise, and process primary sources in the same way that primary sources are described, discussed, and processed [19]. Secondary source sources include pieces in newspapers or popular periodicals, book or movie reviews, and articles published in scientific journals that debate or critique someone else's original study. The usage of secondary source material is beneficial in identifying a diversity of viewpoints and possibilities that may be relied upon either for substantiating or aiding refutation of the arguments in the Secondary Source Method. It is a purely theoretical way of doing research.

### 2.1. Content-Based Image Recovery

CBIR has a wide range of applications and is important in a wide range of fields, including military relations, medical research, education, architectural architecture, the justice department, and agriculture, because photographs contain a wealth of information and have no language restrictions, which encourages international trade, among other things. A number of CBIR systems have evolved throughout the years. The following are some examples of CBIR retrieval schemes: QBIC, Virage, picture book, visual search, NETRA, and simplicity, to name a few.

CBIR is an acronym that refers to image data that has been particularly obtained by looking at images that have specified qualities or that include unique information from an object archive [21]. The core premise of CBIR is the study and use of feature vectors as pictures indexes to understand image information utilising low-level properties, such as colour, texture, shape, and spatial reference objects, among other things. Retrieval approaches, which are generally focused on an image's multidimensional features, are concerned with retrieving images that are similar to each other. The phrase “stuff” in this context refers to the colours, forms, surfaces, or other materials that might arise from the real thing. CBIR is beneficial since most picture search engines on the Internet concentrate primarily on metadata, resulting in a large amount of trash [22].

**Figure 1:**
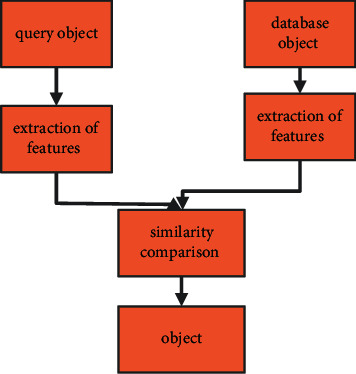
Content-based image recovery block diagram.

Humans may also be inefficient, expensive, and incapable of manually collecting all keywords characterizing the topic for items in a large database when working with large datasets. A more reliable indexing and returning result to a device that can sort objects according to their content [23] will be part of this. In the image retrieval based on content, just the texture, colour, and shape of the objects are employed to identify them as shown in Figure 1.

### 2.2. Text-Based Image Recovery

**Figure 2:**
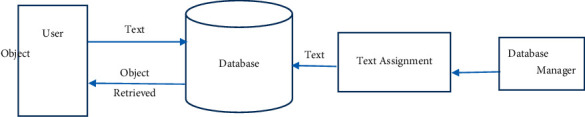
Text-based image retrieval.

It is possible to recover content-based images using the process of embedding information, such as watchwords, endorsements, or image representations, inside the image content. Due to the recovery employed over the annotation words, the annotation process is tough and time-consuming, and it requires a significant amount of work to manually annotate the photographs. In the case of TBIR, the semantically zed text is not present [24]. Using an immersive image recovery technique combined with user term input as shown in Figure 2, we may gather words from all sectors, increasing ambiguity while also increasing the likelihood of generating unrelated concepts [5].

### 2.3. Retrieval by Colour

The colour-based object retrieval strategy is the most straightforward and fundamental approach for CBIR. The colour properties of a material are the most visually appealing and intuitive to the human eye. It is also an important component of interpretation. The colour qualities of things are very robust and durable when compared to other features of objects such as texture and form [25]. It is impervious to changes in the rotation, translation, or size of the object. Furthermore, the colour function [26] may be calculated with a reasonable amount of ease. A colour histogram is a most often used technique for extracting colours from images.

The geometric shape, the colours, and the texture of the material are the most distinguishing aspects of CBIR. The colour element is one of the most widely used pictures recovery functions, and it is one of the most versatile. Colours are recognized based on the colour space that has been selected. There are a variety of colour spaces available, most of which are intended for different purposes. They are there to help. Use colour to create spaces that are akin to human experience.

## 3. Existing Method

In their present approaches, the writers have used a variety of various methodologies. They have coupled the processed form and item data with shading highlights extracted from the spatially organized L2 standardized coefficients in order to create a more realistic appearance. A fraction of the current techniques uses the SIFT (scale-invariant component change) process, which is a computation that is used to locate and depict close highlights in computer-generated images [27]. Finding specific core concerns and then providing them with quantitative data (referred to as descriptors) that may be used for object recognition are among the applications for which it is suitable.

**Figure 3:**
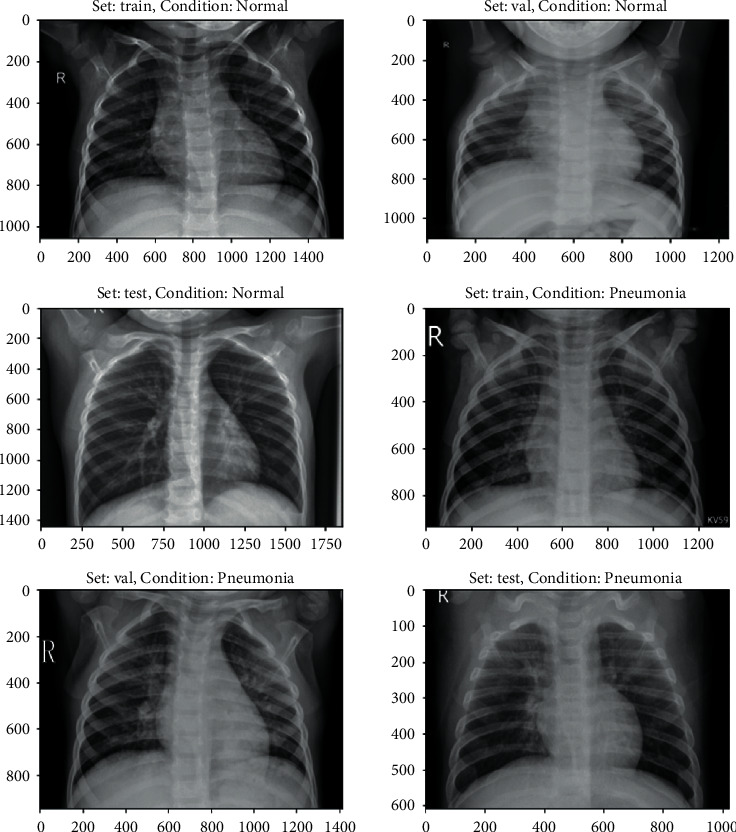
Query object.

When dealing with vast volumes of data, a brute force search is not feasible; thus, more effective search strategies must be used. The bag of features is a straightforward encoding system that uses a small number of visual word histograms to represent a big number of pictures in a little amount of space. This approach, which makes use of an inverted index data structure, enables for small storage while yet providing for excellent search [28]. When it comes to retrieval functions, the kind of items in the database has a lot to do with what is utilised in Figure 3.

A function bag is a technique for extracting the characteristics of an object from its surroundings. According to the classification theory (Word Bag), once numerous representative keywords have been excluded from the object, a dictionary is generated, and an object is calculated based on the number of events that occur in order to get the attribute vector [29]. When it comes to developing a robust vocabulary, a large quantity of data is necessary, which means a large dataset. The most linked cluster centres in the dictionary may be found for each characteristic of the item, and a vector representation of the item called a Bag can be found for each feature of the item by searching for the most connected cluster centres in the dictionary. It should be possible to discern between photos belonging to separate categories in this situation as a consequence of the use of this vector. In order to categorise items, we may utilise this information to train classification models, which we can then use to categorise objects [21].

Afterwards, the features and descriptors for each object are collected, often via SIFT, and mapped into the descriptor space, which varies depending on the descriptor representation used to represent the features in the first place. The image database can be searched to find the most similar visual word in the lexicon for each SIFT function in the picture [30], which we can then use to find the most related visual word. Making a *k*-dimensional histogram of the SIFT function of each individual item in the dictionary may be used to count the items in the dictionary as one method of counting the objects. The Retrieve objects are responsible for returning the IDs of the photos as well as the scores for each result. The outcomes are arranged in decreasing order from the greatest to the most disappointing.

When it comes to accurately identifying locations, we must effectively reflect the features of an item, which can then be grouped together and scanned after they are found to be comparable to one another. Objective recovery may be used to discover objects in things when objects close to the specified object can be readily identified, as well as for other purposes, such as object recovery [31].

**Figure 4:**
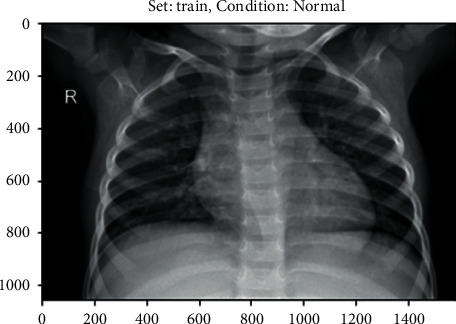
Output for the given query object.

However, Figure 4 represents the present approach is primarily focused on the X-rays and fails to accomplish accuracy in the colour moment, chi-square test, HSV, specificity and sensitivity tests, Gabor wavelet, edge gradient, and retrieval score tests. We were able to accomplish the aforementioned restrictions using the suggested approach.

### 3.1. The Proposed Method

The COVID-19 outbreak has caused a significant increase in online buying activity in recent months. COVID-19 has a moderating influence in the consumer's awareness of services, allowing shoppers to purchase goods and services on the Internet. For the purpose of extracting identical photographs utilising the Bag of Features approach, we recommend that you use an Analogous Product Identification in Healthcare Monitoring service. Training a sequence of random items from the dataset results in the generation of Bag of Features. Afterwards, we index the object that needs to be retrieved, and then we retrieve objects that are identical to the query object that was obtained.

As previously stated, the prior approaches based on picture retrieval had certain limits in terms of accuracy, as well as concerns with the datasets used in their development. As a result, we chose to improve the quality of the datasets while simultaneously making improvements to the datasets themselves in order to overcome the constraints of the prior articles.

The following are the steps to obtain comparable items for shopping: (A) Select the item to be retrieved; (B) create a bag of characteristics for the object to be retrieved; (C) catalogue the item; and (D) look for comparable objects in the index:

- *Select the Item That Will Be Retrieved*.
- When searching for a picture collection that comprises sceneries (beaches, towns, highways, etc.), a global object characteristic, such as a colour histogram that captures the colour contents of the whole scene, is preferred over a more specific object characteristic. If, on the other hand, the aim is to locate certain items in a series of images, it is more practical to use local image attributes that have been retrieved around the important objects.
- *Assemble a Collection of Features in a Bag.*
- By picking a random sample of things from the dataset and then training them on those items, the bag of features may be constructed. In order to train while extracting features, Bag of Feature does not need any associated labels; hence, it is a weakly supervised learning strategy that does not require a significant number of labels. This technique, on the other hand, does not take into consideration the spatial relationship between attributes in any manner whatsoever. The situation is particularly unpleasant when there are numerous potential matches that are suitable, but only one of them is selected because it has a little better score [32]. In such a case, it is critical to develop a genuine method for discriminating between several options in order to determine which is the most suitable one to implement. Providing geometric information in addition to the text may help us avoid this difficulty.
- *Make a Picture Index of All of the Images*.
- To construct a sufficient vocabulary, it is necessary to have a large enough dataset and a large enough quantity of data available. In the next phases, the characteristics and descriptors for each object are extracted and shown. Typically, this is accomplished by mapping into the descriptor space, which is reliant on how the descriptor is expressed in the descriptor space. It is necessary to first discover which visual words are included inside a query object before we can search for those visual words within the query object. Each time a word appears, we look to see whether any further items have the same phrase as the one we are looking for. For each of the items on the ballot, the number of votes in the voting array [22, 33] is raised by one. As seen in the example below, each item in the voting array is a list with one entry for each object that has a counter variable. At the completion of the procedure, we choose the item in the voting array that has the biggest counter value as the match for the query object from among the items in the voting array. However, every attribute in the query object must still be compared to each and every visual word in the vocabulary, even if the query object contains no visual terms.
- *Consider Items That Are Similar to Yours*.
- The next step is to make use of the get objects function in order to find things that are similar to the ones you have already discovered in the previous stage.

It has been discovered that there are some limitations to the accuracy of picture retrieval, and that only a few datasets are used in the present literature when it comes to image retrieval. In order to achieve a Healthcare Monitoring application, we have overcome these limitations by increasing the accuracy of retrieving objects that are similar to the query object, as well as by adding datasets that will assist customers in purchasing the items that they require, increasing the purchase rate, and encouraging customers to search for additional products.

**Figure 5:**
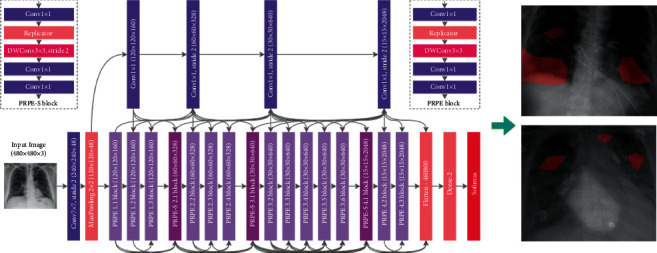
X-ray dataset.

Choosing the photo query that will be returned is the first step (i.e., input object). To be able to get images from the dataset for inclusion in the sample, he must utilise the query picture in the role of a user image. Source constraints do not apply to the picture query, and the image does not have to be from our dataset in order to be valid. As soon as the query image shown in Figure 5 is entered into the computer, the computer snaps a photo of it.

**Step 1 .**

With the feature type defined, the next step is to learn the visual vocabulary within the bag of features using a set of training products. The bag of features is created by picking some random set of products from the dataset and by training them using “Custom Extractor” option, which is used to take out features from each product. Now that the bag of features is created, the entire X-ray image set can be indexed for search.

**Step 2 .**

**Figure 6:**
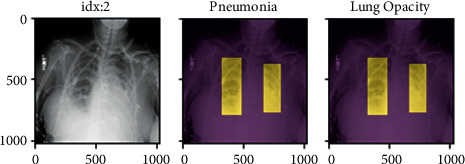
Index object.

The entire X-ray picture collection can now be indexed for searching, until the feature bag is generated. The indexing process extracts features from each product using step 1 personalized extractor function.  Figure 6 represents the features extracted are encoded and added to the product index in a graphic word histogram.

**Step 3 .**

**Figure 7:**
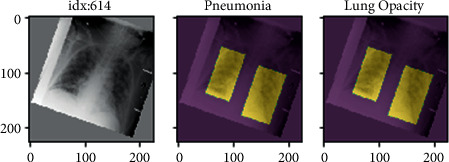
Output X-ray objects.

The final step is to use the retrieve images function to search for similar images. Thus, the accuracy of objects was met by output 1 in Figure 7.

**Figure 8:**
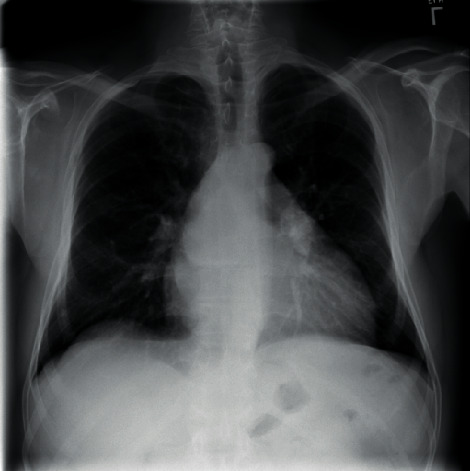
Index object.

**Figure 9:**
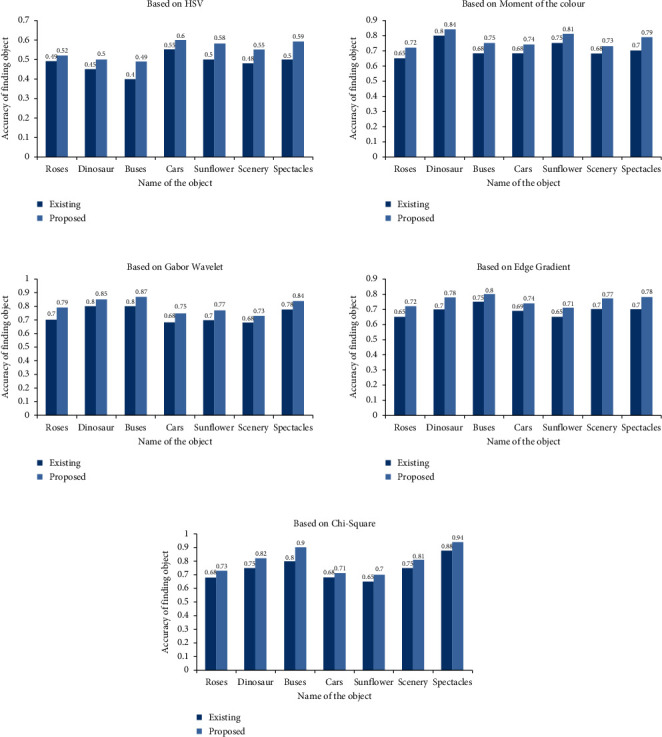
Comparison of different techniques.

Figure 8 shows another index object. The output of this object is shown in Figure 9.

Thus, we added other objects like toys, sceneries, buses, and so on in the dataset other than X-rays as an output.

## 4. Results and Analysis

### 4.1. Results of Examination of Different Techniques

Table 1 values are plotted and the proposed method values are compared with the existing methods.

According to the above graph in Figure 9, the object detection accuracy increased by 7 percent in the proposed method when compared to the HSV method, 6 percent when compared to the colour moment method, 7 percent when compared to the Gabor wavelet method, 6 percent when compared to the edge gradient method, and 6 percent when compared to the chi-square method, respectively.

Methods that are currently in use include HSV (Hue Saturation Value).

In the HSV, we utilised hue to differentiate between the colours, saturation to add white lights to pure colour, and value to detect the strength of light in the image. A colour histogram depicts the distribution of colours in a particular object. According to the findings of this research, the colour properties of an image were used to construct a vector for the categorization of photographs. It performs well in certain data formats while performing poorly in others. HSV, or Hue Saturation Value, is a technique for distinguishing between image brightness and shading data. The use of this method is more convenient when dealing with or requiring brightness of the things [20].

#### 4.1.1. The Colour of the Moment

Based on the colour features that have been proved to define the colour distribution of an item, we used the colour moment to distinguish between photographs from the dataset that were similar in colour.

A colour moment was used on colour photos in the existing project, and the Local Binary Pattern was used to create a textural element, but the shape features were not taken into consideration [34]. Colour moments are computed by taking the mean, the standard deviation, and the skewness of an item and multiplying them together.

### 4.2. Gabor Wavelet

This is a wavelet that was developed by Gabor himself.

This parameter was used to extract texture from an item in order to retrieve it, and it was shown to be incredibly effective. Essentially, a wavelet caught energy at a certain frequency and orientation, and it was found to be extremely efficient. The colour-based CBIR technology incorporates two extra features that are computed using the Gabor wavelet to apply the texture feature, despite the fact that retrieval time is becoming more and more inefficient with each passing generation [22, 35], it is used to extract texture from objects so that it may be retrieved later.

#### 4.2.1. Edge Gradient

We utilised this option to ensure that our feature and texture matching was as robust as possible. In this case, it informs us about the abrupt variations in picture brightness that have occurred. Because of the rise in noise, the gradient magnitude of the edges worsens as well, leading to imprecise results in the current research, which employs Sobel operators for edge detection [36]. The edge detection block locates edges by searching for the local maximum of the gradient of the input object in the surrounding area.

#### 4.2.2. The Value of the Chi-Square Test

Using the chi-square approach, we have been comparing observed data with projected outcomes. They are designed to determine if a disparity is the result of an accident or a relationship between the elements that affect the actual data and what is anticipated by computer models [37]. In the current study, they used a chi-square analysis to match photos [26], although this method does not work for all of the photographs in the collection, which is problematic. When comparing predicted and actual outcomes, it is utilised to determine the difference in size between the two [19].

The attributes that are observed are those that we have accumulated ourselves. The predicted values are calculated based on our null hypothesis, which describes how the anticipated frequencies should be distributed.

**Figure 10:**
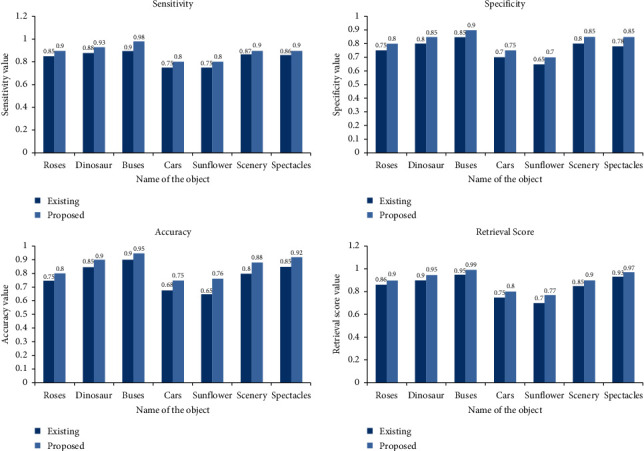
Comparison of different parameters.

Figure 10 shows the comparison of different parameters.

#### 4.2.3. Parameters

*(1) Sensitivity*. This parameter is used to find the value of number of objects is correctly matched. It is calculated by

(1) $$\begin{array}{c} \text{sensitivity}=\frac{\text{TP}}{\left(\text{TP}+\text{FN}\right)}, \end{array}$$

where TP is True Positive, which means the number of matched products that are correctly identified and FN is False Negative, which means number of matched products that are not correctly identified.

*(2) Specificity*. This parameter is used to find the value of number of products that are not matched. It is calculated by

(2) $$\begin{array}{c} \text{Specificity}=\frac{\text{TN}}{\left(\text{TN}+\text{FP}\right)}, \end{array}$$

where TN is True Negative, which means number of products that are not matched and those are correctly identified and FP is False Positive, which means the number of nonmatched products that are not correctly identified.

*(3) Accuracy*. It gives us the average of sensitivity and specificity and is calculated as

(3) $$\begin{array}{c} \text{AC}=\frac{\left(\text{sensitivity}+\text{specificity}\right)}{2}. \end{array}$$

*(4) Retrieval Score*. It is computed for each query product. Its formula is given by

(4) $$\begin{array}{c} \text{retrieval score }=\;100*\left[1-\left(\frac{\text{mismatches}}{\text{n}}\right)\right]\text{\%,} \end{array}$$

where *n* is the number of closest product to the query product.

Table 1 provides experimental results obtained using traditional methodologies and the suggested technology, respectively. This employment contributes to the reduction of a person's burden. With the use of an algorithm, a computer may be taught to recognize different types of photographs. When a computer extracts features from an image, just the collection of features is saved, and only that feature set is sufficient when the picture satisfies the criteria. When you extract characteristics, they are no longer required.  Table 2 shows the exact parallel of the proposed process to the existing procedure. It demonstrates that the precision of the technology being offered has increased.  Table 2 shows the values of the various parameters that may be derived from the outcome of the experiment.

## 5. Conclusion and Future Work

Using this gadget, you may connect a purchasing portal to a knowledge platform. Through this machine, the client will automatically scan for comparable photographs from the information portal in accordance with his favourite images that he has saved. Last but not least, the purchasing ratio of consumers would improve. In addition, the gadget will automatically acquire information from the other Healthcare Monitoring site, give customers the same image recovery straight from the device, and display the search results in a personalized way. Consequently, by adopting the abovementioned approach, the HSV, colour moment, Gabor wavelet, edge gradient, chi-square, sensitivity, specificity, accuracy, and retrieval scores will be raised by 7%, 6%, 7%, 6%, 6%, 4%, 5%, 7%, and 4%, respectively.

## Figures and Tables

**Table 1:** Comparison with different objects and different methods.

Name of the object	HSV method		Moment of the colour method		Gabor wavelet method		Edge gradient method		Chi-square method	
	Exis.	Pro.	Exis.	Pro.	Exis.	Pro.	Exis.	Pro.	Exis.	Pro.
Roses	0.49	0.52	0.65	0.72	0.7	0.79	0.65	0.72	0.68	0.73
Dinosaur	0.45	0.5	0.8	0.84	0.8	0.85	0.70	0.78	0.75	0.82
Buses	0.4	0.49	0.68	0.75	0.8	0.87	0.75	0.8	0.8	0.9
Cars	0.55	0.6	0.68	0.74	0.68	0.75	0.69	0.74	0.68	0.71
Sun X-ray	0.5	0.58	0.75	0.81	0.7	0.77	0.65	0.71	0.65	0.7
Scenery	0.48	0.55	0.68	0.73	0.68	0.73	0.7	0.77	0.75	0.81
Spectacles	0.5	0.59	0.7	0.79	0.78	0.84	0.7	0.78	0.88	0.94
Avg. accuracy	0.47	0.547	0.68	0.768	0.75	0.8	0.7	0.757	0.76	0.801

**Table 2:** Experimental results for parameters of proposed system.

Name of the object	Sensitivity		Specificity		Accuracy		Retrieval score	
	Exis.	Pro.	Exis.	Pro.	Exis.	Pro.	Exis.	Pro.
Roses	0.85	0.9	0.75	0.8	0.75	0.8	0.86	0.9
Dinosaur	0.88	0.93	0.8	0.85	0.85	0.9	0.9	0.95
Buses	0.9	0.98	0.85	0.9	0.9	0.95	0.95	0.99
Cars	0.75	0.8	0.7	0.75	0.68	0.75	0.75	0.8
Sun X-ray	0.75	0.8	0.65	0.7	0.65	0.76	0.7	0.77
Scenery	0.87	0.9	0.8	0.85	0.8	0.88	0.85	0.9
Spectacles	0.86	0.9	0.78	0.85	0.85	0.92	0.93	0.97
Average	0.85	0.882	0.78	0.814	0.83	0.851	0.86	0.897

## Data Availability

The data that support the findings of this study are available upon request to the corresponding author.
